# Personalized Deep Bi-LSTM RNN Based Model for Pain Intensity Classification Using EDA Signal

**DOI:** 10.3390/s22218087

**Published:** 2022-10-22

**Authors:** Fatemeh Pouromran, Yingzi Lin, Sagar Kamarthi

**Affiliations:** Department of Mechanical and Industrial Engineering, Northeastern University, Boston, MA 02115, USA

**Keywords:** pain intensity classification, recurrent neural networks, machine learning, deep learning, EDA signal

## Abstract

Automatic pain intensity assessment from physiological signals has become an appealing approach, but it remains a largely unexplored research topic. Most studies have used machine learning approaches built on carefully designed features based on the domain knowledge available in the literature on the time series of physiological signals. However, a deep learning framework can automate the feature engineering step, enabling the model to directly deal with the raw input signals for real-time pain monitoring. We investigated a personalized Bidirectional Long short-term memory Recurrent Neural Networks (BiLSTM RNN), and an ensemble of BiLSTM RNN and Extreme Gradient Boosting Decision Trees (XGB) for four-category pain intensity classification. We recorded Electrodermal Activity (EDA) signals from 29 subjects during the cold pressor test. We decomposed EDA signals into tonic and phasic components and augmented them to original signals. The BiLSTM-XGB model outperformed the BiLSTM classification performance and achieved an average F1-score of 0.81 and an Area Under the Receiver Operating Characteristic curve (AUROC) of 0.93 over four pain states: no pain, low pain, medium pain, and high pain. We also explored a concatenation of the deep-learning feature representations and a set of fourteen knowledge-based features extracted from EDA signals. The XGB model trained on this fused feature set showed better performance than when it was trained on component feature sets individually. This study showed that deep learning could let us go beyond expert knowledge and benefit from the generated deep representations of physiological signals for pain assessment.

## 1. Introduction

Pain intensity assessment is critical to diagnosing, intervention, patient monitoring, and pain management. Current pain intensity assessments are mainly based on patients’ self-reported pain, using tools such as the visual analog scale (VAS) and numerical rating scale (NRS). Patients are asked to report their pain level from “no pain” to “severe pain” or on a scale of 0 to 10. However, these methods have severe limitations when patients cannot validly communicate their pain intensity, especially infants, toddlers, or adults with mental or other disabilities. Often, these self-reported pain scores are biased when patients exaggerate their pain level due to emotional distress or drug-seeking behavior. Furthermore, these methods give point-in-time measurements and are not practical for continuous pain monitoring. Hence, there is a need to design an objective pain assessment system to objectively measure patients’ pain intensity.

More than ten years after the early studies in automatic pain recognition, modeling and objective estimation of pain intensity remains complex and mostly unexplored. A continually growing number of studies have focused on automatic pain recognition based on machine learning techniques. However, most of these studies proposed pain recognition models based on videos or images from facial expressions and body movements. Recent advances in deep learning for image classification improved the predictive performance significantly. However, these camera-based methods have inherent limitations. They pose privacy concerns, require complex setup, and are not practical for clinical settings and wearable devices. These limitations have motivated researchers to shift their focus from behavioral pain expressions to physiological pain responses as pain biomarkers. They found some evidence that alteration in physiological signals in response to pain can be helpful in objective pain assessment. These physiological signals include Electrocardiogram (ECG), heart activity; Electromyogram (EMG), muscle activity; Electrodermal Activity (EDA), sweat-gland activation, often referred to as skin conductance (SC), and galvanic skin response (GSR); Respiration (RSP); Electroencephalogram (EEG), the electrical activity of the brain; Photoplethysmogram (PPG), blood perfusion of the skin for a pulse and other measures, also called blood volume pulse (BVP).

The current work focuses on designing end-to-end deep learning personalized models for pain intensity estimation and pain level classification tasks for nociceptive cold pain using EDA sensor. Unlike the previous machine learning models, which rely on a hand-engineered set of features extracted from signals, we use complex features extracted from the raw EDA signal by pre-final layers of deep recurrent neural networks. The end-to-end architecture of automated pain assessment helps us go beyond expert knowledge and prepare for real-time pain monitoring. We also built a traditional machine learning model based on the EDA signal features to compare the model performances. We explored the effect of concatenating the features selected based on the domain knowledge available in the literature with the deep learning feature representations. The paper structure is as follows: Section 2 reviews the related works. Section 3 portrays the overall architectures of the proposed models. Section 4 presents the overall results of each model. The article ends with a conclusion in Section 5.

## 2. Related Works

Some research teams have built databases of physiological signals generated in response to pain. For example, *BioVid Heat Pain Database* [1,2] provides multimodal sensor data—different physiological signals and videos—in response to short-term painful heat stimuli. Several studies have explored this database’s EDA, ECG, and EMG signals to build machine learning models for pain classification or pain intensity estimation tasks [3,4,5,6]. These automated pain recognition models have mainly used a set of carefully selected features based on the domain knowledge available in the literature on time series of raw physiological signals. The most common features extracted from physiological signals are statistical time-domain or frequency-domain features calculated on the raw signals after minor preprocessing and normalization. They perform feature selection to reduce the dimensionality of data and train a machine learning model based on those selected features. Support Vector Machines (SVMs) and Random Forests are the most frequently used algorithms for pain intensity estimation. Susam et al. [7,8] used timescale decomposition (TSD), a measure of simultaneous measuring long and short-term changes in time-series data, for statistical feature extraction from EDA signals. They built a linear SVM-based model for binary pain classification in children across the two visits during the recovery period following laparoscopic surgery. With the advent of deep learning, Martinez et al. [9] explored the application of Recurrent Neural Networks (RNN) for automatic pain intensity estimation on the BioVid dataset. They showed that the RNN model on the knowledge-based extracted features from the EDA signal outperforms the traditional machine learning algorithms. Thiam et al. [10] used the *SenseEmotion Database* [11] which presents measurements collected from healthy participants when they were subjected to a series of artificially induced heat pain stimuli. They recorded several signals, including audio, video, EMG, ECG, RSP, and EDA, and explored single modality and multimodal pain assessment models based on different data fusion strategies. Readers can find a good overview of the automatic pain assessment methods in Werner et al. [12] and Wagemakers et al. [13].

The application of deep learning methods to physiological signals has recently received increasing attention among researchers but the benefits of this novel approach have not been fully explored [14]. Traditionally, machine learning models based on physiological signals have primarily relied on features selected based on the domain knowledge available in the literature. Still, these manual features may not capture the complete information embedded in the raw signal. Manual feature extraction and feature selection from physiological signals are time-consuming, suboptimal, inflexible, limited to the expert’s knowledge and reasoning, and not suitable for real-time monitoring. However, deep learning models automatically perform the feature extraction and feature selection within multiple hidden layers, from raw input signals or low-level processed signals. An increasing number of studies have been exploring deep learning applications on physiological signals, and most of them are on EEG signals. Ganapathy et al. and Fawaz et al. [15,16] provided a good overview of deep learning models on 1-D bio-signals and time series. However, only a few papers were published on deep learning models for pain recognition using physiological sensors [17]. Yu et al. [18] proposed various frequency bandpass-based CNNs for subject-dependent cold pain state classification using EEG signals. Thiam et al. [19,20] explored CNN-based models for pain recognition using EDA, EMG, and ECG signals from the BioVid heat pain dataset. Subramaniam et al. [21] built a hybrid CNN-LSTM model on EDA and BVP signals from the BioVid dataset for binary pain recognition.

EDA has been proven to be the best performing single modality for pain classification, better than Video, ECG and EMG, and RS signals. Studies report that EDA is less sensitive to the individual characteristics of each participant [6,10,19,22,23]. The other advantage of using EDA is that it can be easily measured using a wrist sensor, making it an excellent modality for the wearable pain monitoring device. One of the essential aspects to consider in the pain-sensing system is the setup complexity. Although the inclusion of a large number of different signals may boost the predictive power of a model, the approach becomes impractical for real-world applications. One of the ideal pain-sensing systems would be the one that can fit with smartphones and fitness trackers [5,24]. For these reasons, an automatic end-to-end pain assessment system based on an EDA signal can enable the integration of the system into wearable devices for online pain intensity recognition or pain monitoring in clinical settings.

EDA signal has shown some promising performances in other domains such as automated emotion recognition [25,26,27], depression disorder detection [28], stress detection [29,30,31], and sleep. Karen et al. [32] applied a series of CNNs and RNNs for emotion recognition using EDA and ECG signals directly from the raw representation. Using the RECOLA database, they showed that this end-to-end learning approach yields a considerable improvement in pain assessment over the knowledge-based features. They focused on EDA and ECG because these signals can be easily captured with wearable devices such as smartwatches and smart bracelets. In the preprocessing phase, they performed down-sampling, normalization, and windowing of the input signal. To augment the training examples, they extracted the maximum number of overlapping windows from each signal (windows were shifted by one timestep from each other), paired with the annotation corresponding to the window’s center. They employed a late fusion scheme with linear regression. Huang et al. [33] proposed an emotion classifier model using an ensemble of five Convolutional Neural Networks (CNN) and a global average pooling layer instead of a fully connected layer. They combined 32-channel EEG signals with three peripheral physiological signals, including EDA, Respiration Belt (RB), and Electrooculogram (EOG). They used the DEAP dataset in which each of 32 participants watched 40 one-minute-long excerpts of music videos and rated the levels of arousal, valence, like/dislike, dominance, and familiarity. They randomly chose 16 subjects from the DEAP dataset for the analysis. Machot et al. [34] proposed a CNN architecture on raw EDA signals for emotion classification. The CNN architecture had three convolution layers, each followed by a pooling layer, and the final output layer followed by two fully connected layers. This assessment selected 10 subjects from the DEAP and MAHNOB dataset, consisting of 4 classes for each subject.

Recurrent Neural Networks (RNNs) provided state-of-the-art health monitoring results using physiological signals. In sleep quality monitoring, RNN-based models were utilized to automatically detect sleep-disordered breathing events and classify sleep stages using ECG recordings [35]. These models were also used for detecting negative respiratory events during sleep using polysomnography signals [36,37]. In mental health, recurrent models have been applied on EEG for emotion monitoring [38] and mental disorder diagnosis [39]. In heart patient monitoring, the LSTM-based model was used on ECG signals to detect congestive heart failure [37] and classify heart disease [40]. Moreover, recurrent neural networks have been used for blood pressure monitoring from ECG and PPG signals [41,42].

## 3. Materials and Methods

### 3.1. Cold Pain Experiment

The data were collected through a cold pain experiment conducted at the Intelligent Human–Machine Systems Laboratory, according to the study protocol approved by the Northeastern University Institutional Review Board (IRB No. 191215, Approval date: 20 December 2019). Twenty-nine healthy subjects participated in this experiment. Per the protocol, consent to participate in the study was sought from all participants. For each subject, the experiment was repeated three times, each session on a weekday of three different weeks. During the experiments, the data were collected from multiple sensors attached to the subject, but within the scope of this paper, we are describing the measurement of EDA only. EDA reflects changes in the skin’s electrical conductivity due to the activation of sweat glands, which are controlled by the Autonomic Nervous System (ANS). The skin conductance was measured using FlexComp Infiniti EDA sensor, which has two probs. One of the probes was attached to the subject’s index finger and the other to the ring finger. FlexComp Infiniti is a physiological monitoring and biofeedback system produced by Thought Technology, Canada. At the beginning of each experiment, the subject was asked to relax and focus on a green dot displayed on a monitor in front of the subject, and a 20-s recording was taken from the EDA sensor to serve as a baseline measurement. After the baseline measurement, the subject was asked to immerse his/her hand into the bucket of iced water continuously for 220 s during which period the EDA signal was collected at 2048 Hz sampling frequency. The subject was advised to withdraw his/her hand from the cold water at any time when he/she cannot bear the pain. While the hand is in the cold water continuously, the subject was asked to report every 20 s his/her pain level on the 0 to 10 verbal rating scale (VRS), where 0 being no pain, and 10 being the most painful. This reported pain level, say at time *t*, was used for labeling 10 windows before *t* and 10 windows after *t*, considering 1 s as the window length. The experiment ended after a total of 10 sessions or any time the subject wanted to stop. The sampling frequency of 2 kHz is commonly recommended in the literature for EDA signals [43]. Lin et al. [44] generated the data in the current study at a 2048 Hz sampling rate. A comprehensive description of the experimentation is available Lin et al. [44].

### 3.2. Data Preprocessing

In this study, the EDA signal for each subject was first filtered by applying a third-order Butterworth low-pass filter with a cutoff normalized frequency of 0.7 Hz to remove noise and artifacts. It is a common practice to decompose EDA signals into tonic and phasic components [45]. The tonic component, known as Skin Conductance Level (SCL), represents a slowly varying conductivity baseline. On the other hand, the phasic component, known as Skin Conductance Response (SCR), presents peaks in the EDA signal. Literature offers multiple approaches to decomposing EDA signals. The approaches include the convex optimization approach [46] and the generalized-cross-validation-based block coordinate descent approach [47]. We used the convex optimization technique [45] to decompose EDA signals into the tonic and phasic components and augment them to the original EDA signals. In addition, we segmented the data into the non-overlapping windows of length 2048. Through feature-engineering-based techniques, we extracted knowledge-based features from each window. In Table 1 we presented the set of EDA features extracted from tonic and phasic components of EDA signals. We computed these features using a custom code built on Python’s Pyphysio library functions [48].

**Figure 1:**
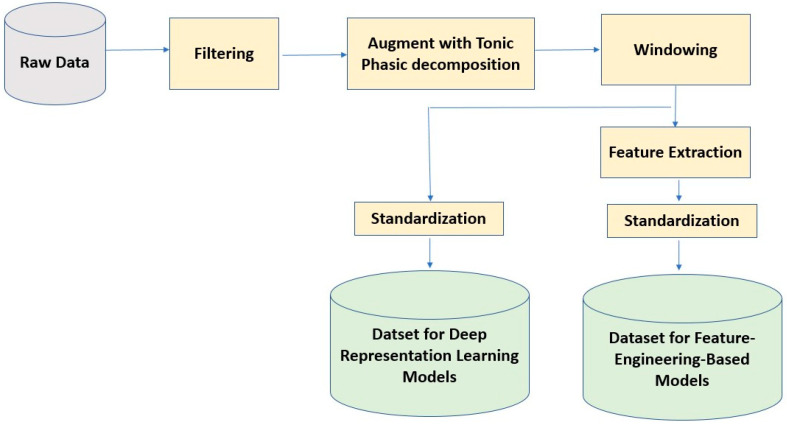
Sensor data preprocessing steps for deep learning and feature-engineering based models.

We standardized each subject’s data to have zero mean and unit variance. This standardization technique helps us mitigate the effect of between-subject variability in physiological signals. For the pain intensity classification task, we categorized the pain scores into four pain states: No Pain (Pain = 0), Low Pain (0 < Pain ≤ 3), Medium Pain (3 < Pain ≤ 7), and High Pain (7 < Pain ≤10). Then, we used this 4-category data to build a deep-representation learning and a feature-engineering-based model for pain intensity classification using the EDA signal. The sensor data preprocessing steps are illustrated in Figure 1.

We conducted stratified sampling to split the data into 80% training and 20% testing sets. Then, we used 20% of the training data as a validation dataset for hyperparameter tuning and the rest for training. Since the data was imbalanced, we applied Synthetic Minority Oversampling Technique (SMOTE) [49] technique on the training dataset to oversample the minority classes. In this technique, we oversample the minority class by creating synthetic examples rather than by resampling with replacement. SMOTE first selects a minority class instance at random as X_1_ finds its k nearest minority class neighbors. Then, randomly chooses one of these neighbors as X_2_. The synthetic instances are generated as a convex combination of these two chosen instances, X_1_ and X_2_. Using this data augmentation technique, we generated as many synthetic examples for the minority class as required to balance the class distribution in the training dataset.

### 3.3. Deep Recurrent Neural Networks (RNN) Based Model

RNNs are a family of neural networks that capture the temporal relationship in sequential data, especially for natural language processing and time-series modeling. RNNs can track previously observed samples by storing the model output in internal memory and passing it as an additional input for the next sample prediction. The network dynamically unrolls on the arrival of new data points to generate a new output using the new input and the last memory state.

Traditional RNN architectures suffer from the vanishing gradient problem during error backpropagation when working with a long sequence of data [50]. If the previous state influencing the current prediction is not in the recent past, the RNN model may not accurately predict the current state. The Long Short-Term Memory (LSTM) [50] network is a variation of RNN originally proposed to tackle this challenge by introducing a novel data forgetting and remembering mechanism. Each LSTM cell contains input, output, and forget gates that jointly control information to be read, stored in the internal memory, or passed to the next cell. Each LSTM cell tracks and updates a “cell state,” or memory variable *C*^〈*t*〉^ at every time step, which can be different from *a*^〈*t*〉^. We begin by implementing the LSTM cell for a single time step. Then, we will iteratively call it from inside a “for loop” to process the input with *Tx* time steps.

The working of LSTM layers is mathematically represented as follows:

(1) $$\Gamma_{i}=\sigma \left(\;W_{i}\;\left[a^{\langle t-1\rangle },x^{\langle t\rangle }\right]+b_{i}\right)$$

(2) $$\Gamma_{f}=\sigma \left(W_{f}\left[a^{\langle t-1\rangle },x^{\langle t\rangle }\right]+b_{f}\right)$$

(3) $$\Gamma_{o}=\sigma \left(\;W_{o}\left[a^{\langle t-1\rangle },x^{\langle t\rangle }\right]+b_{o}\;\right)$$

(4) $${\tilde{C}}^{\langle t\rangle }=tanh\left(\;W_{c}\left[a^{\langle t-1\rangle },x^{\langle t\rangle }\right]+b_{c}\;\right)$$

(5) $$C^{\langle t\rangle }=\Gamma_{i}\;\cdot \;{\tilde{C}}^{\langle t\rangle }+\Gamma_{f}\;\cdot \;C^{\langle t-1\rangle }$$

(6) $$a^{\langle t\rangle }=\Gamma_{o}\;\;\cdot \;\;tanh\;\left(C^{\langle t\rangle }\right)$$

where the input gate $\Gamma_{i}\;$controls input information added to the cell state. The forget gate $\Gamma_{f}$ determines the past information to be retained in the long-term memory. The output gate decides how the regulated information is made available as output.

Bidirectional LSTM (BiLSTM) [51] is an extension to LSTM cells that is shown to improve the performance in many applications. BiLSTM stacks two layers of LSTM cells to process the sequential data in forward and reverse order. In this architecture, the concatenation of the hidden states of the forward and backward layers forms the representation of the sequential data. Processing the data in reverse order is shown to help the model better retain information near the end of the sequence.

We used the Adaptive Moment estimation algorithm (*Adam*) as an optimizer. The model parameters are updated using the backpropagation algorithm. The error between the desired output and the actual output is calculated using the loss function, and the gradient descent method is applied to update parameters to minimize the loss. The mathematical functions to update the weight and bias are shown by

(7) $$W_{k}=W_{k}-\eta \;\frac{\delta E}{\delta W_{k}}$$

(8) $$b_{k}=b_{k}-\eta \;\frac{\delta E}{\delta b_{k}}$$

where $W_{k}$ is a weights matrix; $b_{k}$ is the bias; η represents the learning rate; *E* is the loss. For the multiclass classification task, the loss is calculated by the *Categorical Cross Entropy* function, which is given by

(9) $$loss=-\overset{N}{\underset{n=1}{\sum }}{\hat{y}}_{i1}\log y_{i1}+{\hat{y}}_{i2}\log y_{i2}+{\hat{y}}_{i3}\log y_{i3}+{\hat{y}}_{i4}\log y_{i4}$$

where *N* is the number of samples; $y_{i1}$, $y_{i2}$, $y_{i3}$, and $y_{i4}$ are label values which are one-hot encoded. The four outputs, ${\hat{y}}_{i1}$, ${\hat{y}}_{i2}$, ${\hat{y}}_{i3}$, and ${\hat{y}}_{i4}$ are from the densely connected layer with *softmax* and correspond to the probability score for each pain level.

We built an end-to-end biLSTM Recurrent Neural Network model for pain detection using EDA signals. The size of two BiLSTM layers was chosen among 32, 64, 128, 256 using a grid search on the validation dataset. We downsampled each window to have 30 data points per second by averaging (i.e., dividing 2048 points into 30 segments and taking the average of each segment). So, the wrapped window of the original EDA, and tonic and phasic components had the shape of 30 by 3 matrix. We ran the model for 200 epochs with early stopping criteria to terminate the training when the loss decreased by less than 1 × 10^−7^ on the validation dataset. The learning rate and L_1_ regularization factor were chosen from the range 1 × 10^−1^ to 1 × 10^−6^. The grid search resulted in having two BiLSTM layers of size 64 and L_1_ regularization with a factor of 1 × 10^−4^.

### 3.4. BiLSTM-XGB Model for Multiclass Pain Classification

**Figure 2:**
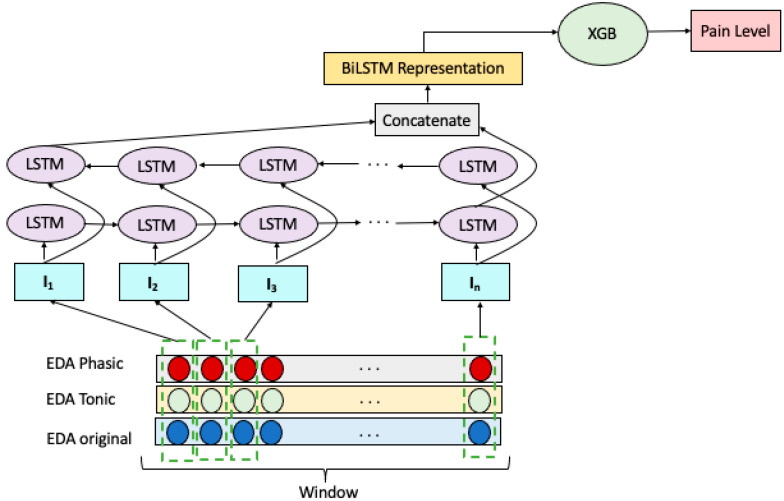
BiLSTM-XGB model for pain intensity classification.

We explored an ensemble of Bidirectional LSTM RNN and Extreme Gradient Boosting (XGB) to build a multiclass pain classification model, as illustrated in Figure 2. We employed a Bidirectional LSTM RNN for extracting representative features of the EDA signals. Decomposing the EDA raw signal into the tonic and phasic components gave us two additional signals of the same length. Therefore, we have three temporally matching windows at each prediction step which are denoted as $W={\left[w_{1},\;w_{2},\;w_{3}\right]}^{T}\;$_._ Each window $w_{j}\;$wraps sensor reading with *n* time steps which represented as: $w_{j}=\left[\;x_{j,1}\;,\;x_{j,2}\;,\;x_{j,3}\;,\;\ldots ,\;\;x_{j,n}\right]{\;}^{T}$. Given these, the input vector to the *i*th LSTM cell of the model is formed by combining the *i*th readings of windows in *W*, forming the vector: $I_{i}=\left[\;x_{1,i}\;,\;x_{2,1}\;,\;x_{3,i}\right]{\;}^{T}$.

The input vectors, ***I***, constructed as above were fed to two layers of LSTM cells, one in the forward direction and the other in the backward order. The concatenation of the hidden states of the last LSTM cells in the forward and backward layers forms the representation of *W*. This representation is then fed to an XGB for the classification of EDA signals as belonging to NP, LP, MP, or HP.

### 3.5. Model Using Knowledge-Based Features Extracted from EDA Signal

We compared the effectiveness of knowledge-based EDA features to the effectiveness of features automatically extracted by the RNN.

### 3.6. Model Using the Concatenation of Deep Representations and Hand-Engineered Features from EDA Signal

We explored the impact of combining the BiLSTM-generated representations with knowledge-based features. In this model, the BiLSTM representation is captured by applying BiLSTM RNN model on the input signals of shape (30, 3), which wraps three signal readings of length 30 timesteps. The three signals are raw EDA, tonic EDA, and phasic EDA. The two BiLSTM layers of size 64 gave us the window representation of length 128 considering the forward and backward flow of bidirectional LSTM. Then, we added the 14 manually extracted features to the LSTEM-provided features. We also built a pipeline to apply the ExtraTree-based feature selection. Finally, we input the selected features to the XGB model for classification into four pain states NP, LP, MP, and HP.

### 3.7. Model Evaluation Metrics

To evaluate the performance of the pain intensity classification model, we used Precision, Recall, and F1 score. The below equations show the expression for these performance metrics in which TP, FP, TN, and FN refer to “True Positives”, “False Positives”, “True Negatives”, and “False Negatives”, respectively. We also employed the Area Under the Receiver Operating Characteristic Curve (AUROC) to evaluate the model performance.

(10) $$\mathrm{Precision}\;=\frac{\mathrm{TP}}{\mathrm{TP}+\mathrm{FP}}$$

(11) $$\mathrm{Recall}\;=\frac{\mathrm{TP}}{\mathrm{TP}+\mathrm{FN}}$$

(12) $$F1=\frac{2\times \;\mathrm{Precision}\;\times \;\mathrm{Recall}}{\mathrm{Precision}+\mathrm{Recall}}$$

## 4. Results and Discussion

We built and tuned different models discussed in this section using Keras [52], TensorFlow [53], and Scikit-learn [54] in Python.

First, we built an end-to-end model using a BiLSTM RNN model using raw EDA signals along with their tonic and phasic components. Explored binary (absence or presence of pain) classification BiLSTM RNN model and four-category (no pain, low pain, medium pain, and high pain) classification BiLSTM RNN models. Table 2 shows the BiLSTM RNN model performance of binary classification. Although the performance of the BiLSTM RNN model on binary classification tasks is good, its performance on the four-category classification task is rather low: the F1 score and AUROC for the four-category model were 0.45 and 0.65, respectively.

We then explored combining the strength of BiLSTM RNN to automatically extract features with the learning ability of XGB. In the hybrid model, we extracted the output of the last BiLSTM layer and used it as an input to the XGB. Table 3 presents the F1 score and AUROC for XGB trained on BiLSTM RNN representations, XGB trained on knowledge-based features, and XGB trained on both BiLSTM RNN representations and knowledge-based features. The four-category XGB model on BiLSTM feature representations gave an average F1 score of 0.81 and AUROC of 0.92. This is a good result with a set of automatically generated representations from a deep learning model on a raw signal. Although the BiLSTM RNN model on raw signal had low model performance, its performance significantly improved when we combined it with the XGB.

In another approach, we trained an XGB model on a set of 14 carefully designed knowledge-based features from EDA signals, which gave us an average F1 score of 0.76 and AUROC of 0.90. This result shows that the XGB model built on the automatically generated feature representations from the BiLSTM achieved superior performance than the XGB model constructed on the knowledge-based features. In the next model, we investigated the effect of concatenating the knowledge-based features with the features from the BiLSTM layer. The results show that this hybrid-feature-based model outperformed the other models with a higher F1 score and AUROC. In other words, we see that we can benefit from augmenting knowledge-based features with temporal dynamic features automatically extracted by the deep RNN model.

The model performances on each pain level in a personalized four-category classification task are reported in Table 4. The classification model results are reported in terms of precision and recall. The model performances are compared in F1 score (the harmonic mean of precision and recall), which is more appropriate than accuracy when the multiclass data is imbalanced.

**Figure 3:**
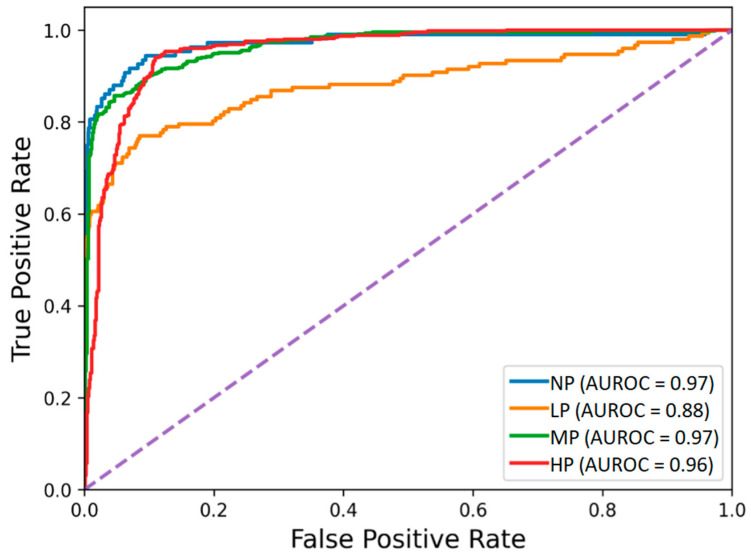
Receiver operating characteristic of the BiLSTM-XGB pain intensity classification model using EDA signal. The four pain categories are No Pain (NP), Low Pain (LP), Medium Pain (MP), and High Pain (HP).

**Figure 4:**
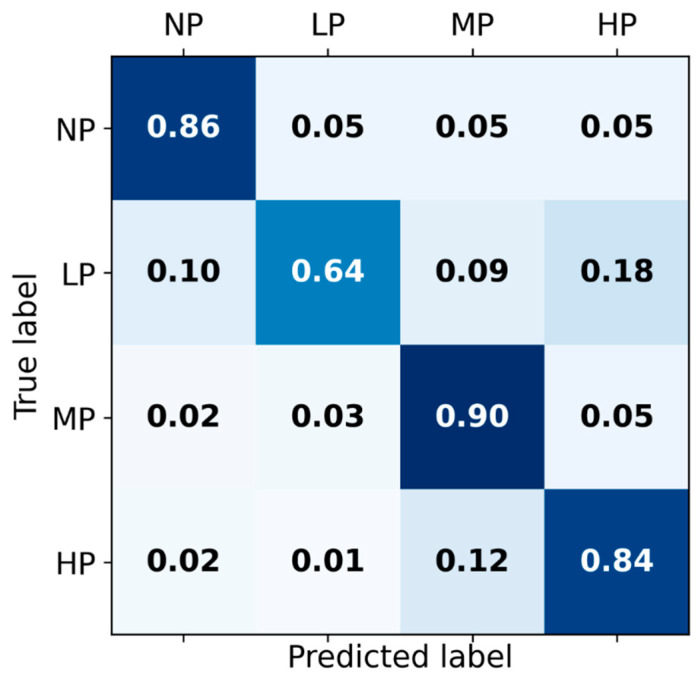
Classification matrix for Hybrid BiLSTM-XGB pain intensity classification using EDA signal among four categories of pain: No pain (NP), Low pain (LP), Medium pain (MP), and High pain (HP) on the test set.

The receiver operating characteristic curves of the hybrid-feature-based model trained on the concatenation of knowledge-based and BiLSTM-based features are depicted in Figure 3. The classification matrix of this hybrid BiLSTM-XGB model is presented in Figure 4.

Although the proposed deep learning framework can provide competitive pain assessment performance, there are some study limitations. This study was conducted on a small number of subjects. In future studies, we will collect data from a relatively large number of subjects. We will explore data processing and feature extraction with a larger signal window. In the current study, all subjects in the experiment were healthy and free of pain; the results might differ if subjects were already in pain due to disease, injury, or other reasons. The proposed models were evaluated on only nociceptive cold pain (due to stimulation of sensory nerve fibers) and did not cover neuropathic pain (due to the impaired somatosensory nervous system) or psychogenic pain (caused, increased, or prolonged by mental, emotional, or behavioral factors). This study did not explore other types of pain such as those stimulated by heat, chemical, or electrical pain inducers. In future studies, we will explore whether body signals have different responses to different types of pain and how a pain intensity estimation model built for a certain type of pain can be applicable to others.

## 5. Conclusions

This study proposed end-to-end deep learning personalized models for automated pain intensity classification using EDA signals. A total of 29 subjects were recruited to participate in the cold pain experiments. The EDA signals, collected from the participants, were used to build the proposed models. Unlike the traditional machine learning models that rely on carefully designed knowledge-based signal features, we let the deep learning architecture automatically find the raw input signal features. This approach allowed the model to augment expert-knowledge-based features with deep-learning extracted features.

The proposed Bidirectional LSTM RNN model learns the temporal dynamics from the raw EDA signals and their tonic and phasic components. We harnessed the power of XGB decision trees on top of the BiLSTM RNN layers for multiclass pain classification to build a model with superior performance. The use of the ensemble of BiLSTM RNN and XGB has given a good classification performance. To our knowledge, this is the first study that used an ensemble of BiLSTM RNNs and XGB for multiclass pain intensity classification using raw EDA signals and their tonic and phasic components. Building XGB with deep-learning-based features improved the model classification performance considerably. This approach taps into the information that the other algorithm cannot access. We compared the BiLSTM-XGB model’s performance with that of the model built on 14 knowledge-based features widely used in the literature. We found that the XGB model receiving BiLSTM-generated features gave better performance than XGB built on knowledge-based features. This shows the ability of deep recurrent neural networks to augment expert knowledge. In addition, we explored concatenating the BiLSTM layer with knowledge-based features and found that they can complement each other to improve the XGB model performance for pain intensity classification.

Overall, this work contributed to the ongoing efforts to automatically quantify perceived pain levels based on EDA signals with an end-to-end automated feature engineering using a deep learning framework. Using minimally preprocessed EDA signals, which a wrist sensor can easily capture, one can build an automated pain monitoring wearable device.

## Figures and Tables

**Table 1:** Tonic and phasic features extracted from EDA signal.

EDA Component	Features
Phasic	Mean, Standard deviation, Range, Peaks Max, Peaks Min, Peaks Sum, Peaks Num, Duration Mean, Slope Mean, AUC
Tonic	Mean, Standard Deviation, Range, AUC

**Table 2:** Performance of BiLSTM RNN binary (presence or absence of pain) classification model.

Classification Task	Precision	Recall	F1 Score	AUROC
NP vs. LP	0.70	0.85	0.77	0.70
NP vs. MP	0.91	0.84	0.88	0.83
NP vs. HP	0.91	0.87	0.89	0.81

**Table 3:** Comparing the performance of four-category pain intensity classification models.

Model	F1-Score	AUROC
XGB on BiLSTM RNN representations	0.81	0.92
XGB on knowledge-based features	0.76	0.90
XGB on BiLSTM RNN representations and knowledge-based features	**0.84**	**0.95**

**Table 4:** Performance of four-category XGB-based classification models.

Model	Pain State	Precision	Recall	F1-Score
XGB on BiLSTM RNN representations	NP	0.69	0.83	0.75
	LP	0.67	0.59	0.63
	MP	0.81	0.87	0.84
	HP	0.84	0.76	0.80
XGB on knowledge-based features	NP	0.68	0.77	0.72
	LP	0.67	0.61	0.63
	MP	0.79	0.77	0.78
	HP	0.78	0.80	0.79
XGB on the concatenation of BiLSTM representations and knowledge-based features	NP	** 0.72 **	** 0.86 **	** 0.78 **
	LP	** 0.82 **	** 0.64 **	** 0.72 **
	MP	** 0.85 **	** 0.90 **	** 0.88 **
	HP	** 0.87 **	** 0.84 **	** 0.86 **

The performance metrics for the four-category pain classification model with four pain states No Pain (NP), Low Pain (LP), and Medium Pain (MP) and High Pain (HP). The best model performances are depicted in bold.

## Data Availability

All data used in this study are managed and protected under IRB No. 191215 dated 20 December 2019.
